# Effect of Octreotide on Hepatic Steatosis in Diet-Induced Obesity in Rats

**DOI:** 10.1371/journal.pone.0152085

**Published:** 2016-03-22

**Authors:** Mao Li, Ting Ye, Xiao-Xia Wang, Xian Li, Ou Qiang, Tao Yu, Cheng-Wei Tang, Rui Liu

**Affiliations:** 1 Division of Peptides Related to Human Disease, State Key Laboratory of Biotherapy, West China Hospital, Sichuan University, Chengdu, China; 2 Department of Gastroenterology, West China Hospital, Sichuan University, Chengdu, China; East Tennessee State University, UNITED STATES

## Abstract

**Background:**

Non-alcoholic fatty liver disease (NAFLD) caused by liver lipid dysregulation is linked to obesity. Somatostatin (SST) and its analogs have been used to treat pediatric hypothalamic obesity. However, the application of such drugs for the treatment of NAFLD has not been evaluated.

**Objective:**

This study aimed to investigate the expression levels of important regulators of hepatic lipid metabolism and the possible effect of the SST analog octreotide on these regulators.

**Methods:**

SD rats were assigned to a control group and a high-fat diet group. Obese rats from the high-fat diet group were further divided into the obese and octreotide-treated groups. The body weight, plasma SST, fasting plasma glucose (FPG), insulin, triglyceride (TG), total cholesterol (TC), low-density lipoprotein cholesterol (LDL-C), high-density lipoprotein cholesterol (HDL-C) and free fatty acid (FFA) levels were measured. Hepatic steatosis was evaluated based on the liver TG content, HE staining and oil red O staining. The SREBP-1c, ACC1, FAS, MTP, apoB and ADRP expression levels in the liver were also determined by RT-PCR, qRT-PCR, western blot or ELISA.

**Results:**

The obese rats induced by high-fat diet expressed more SREBP-1c, FAS and ADRP but less MTP protein in the liver than those of control rats, whereas octreotide intervention reversed these changes and increased the level of apoB protein. Compared to the control group, obese rats showed increased liver ACC1, SREBP-1c and apoB mRNA levels, whereas octreotide-treated rats showed decreased mRNA levels of apoB and SREBP-1c. This was accompanied by increased body weight, liver TG contents, FPG, TG, TC, LDL-C, FFA, insulin and derived homeostatic model assessment (HOMA) values. Octreotide intervention significantly decreased these parameters. Compared to the control group, the obese group showed a decreasing trend on plasma SST levels, which were significantly increased by the octreotide intervention.

**Conclusion:**

Octreotide can ameliorate hepatic steatosis in obese rats, possibly by decreasing hepatic lipogenesis and increasing TG export from hepatocytes.

## Introduction

Non-alcoholic fatty liver disease (NAFLD) is closely related to diabetes and obesity [1, 2] and has become a primary disease endangering public health. The incidence of NAFLD is reportedly 19.0% in the United States and as high as 44.3–56.6% in obese individuals with a BMI (body mass index) exceeding 35 kg/m^2^ [3]. NAFLD is manifested by the accumulation of lipids in the liver in the absence of excess alcohol consumption, caused by the imbalance between lipid input (fatty acid uptake and de novo lipogenesis) and output (VLDL export and fatty acid β-oxidation) [4].

Acetyl-CoA carboxylase (ACC1) and fatty acid synthase (FAS) are the regulatory enzymes for hepatic lipid synthesis. Insulin regulates the transcription and activation of liver sterol regulatory element binding protein-1c (SREBP-1c), which participates in liver fatty acid synthesis by stimulating the expression of ACC1 and FAS. Hyperactivation of SREBP-1c induces hepatic lipid accumulation [5, 6, 7], indicating that SREBP-1c-associated de novo lipogenesis is an important target of hepatic steatosis.

For hepatic lipid export, triglyceride (TG) export depends not only on the secretion rate of VLDL but also on the TG content of VLDL. Microsomal triglyceride transfer protein (MTP) is a rate-limiting factor for the assembly of VLDL, which involves the transfer of a few lipids to apolipoprotein B (ApoB) [8, 9]. The esterification and maturation process of VLDL is associated with adipose differentiation-related protein (ADRP). ADRP can reduce the TG content of VLDL and promote fatty liver disease by diverting fatty acids from the VLDL assembly pathway into the production of cytosolic triglycerides [10].

Octreotide, a synthetic and eight-peptide analog of somatostatin (SST), has a strong affinity to somatostatin receptor 2 (SSTR2), which was high-expressed in the liver. Valuable results show that octreotide has been used to treat obese patients with insulin hypersecretion [11] and pediatric hypothalamic obesity [12]. Recently, we have found that octreotide shows new and important functions in decreasing body weight via the suppression of intestinal fat absorption [13] and also in improving pancreatic fatty infiltration [14] in high-fat diet (HFD)-induced obese rats. However, the effects of octreotide on fatty liver disease in HFD-induced obese rats remain uncertain.

In this study, we determined whether octreotide alleviates fatty liver disease in HFD-induced obese rats. To further investigate the underlying mechanism, we assessed the expression of SREBP1c, ACC and FAS which were associated with lipogenesis, and the expression of MTP, apoB, and ADRP which are associated with lipid secretion.

## Materials and Methods

### Animals and experimental designs

Fifty-six healthy, 21-day-old male Sprague-Dawley rats were purchased from the Animal Center of Sichuan University. All rats were housed in individual cages with 12 h light-dark cycles and given free access to water and chow. After 3 days of adjustable feeding at the Animal Center of West China Hospital, the rats were randomly divided into two groups as follows: the first group containing 16 rats was maintained on standard chow (290 kcal/100 g, in accordance with the People’s Republic of China National Standard GB 14924–2001) for 176 days which was set as the normal control group, whereas the second group containing 40 rats was fed high-fat chow (430 kcal/100 g, supplemented with 10% protein, 15% lard, and 10% sucrose). It has reported that such high-fat diet formulation induces obesity in rats [15]. The body weights and body lengths were measured once per week. Then, Lee’s index [body weight (g)^1/3^ × 1,000/body length (cm)] was calculated. At the end of week 24, 32 eligible obese rats with a mean body weight at least 1.4 times heavier than that of the control group were selected from the high-fat diet rats [16] and divided into the obese group and octreotide-treated group, with 16 rats each. Both groups of rats were continuously fed high-fat chow, but the rats in the latter group were administered a subcutaneous injection of octreotide at a dose of 40 μg/kg body weight twice daily for 8 days. In previous clinical studies, it was shown that both a high dosage (60 mg) and low dosage (40 mg) of octreotide administered to patients resulted in similar weight loss in obese adults with insulin hypersecretion, suggesting that a higher dose of octreotide used did not achieve better curative effect in treating obesity [11]. On the contrary, high dose of octrotide could damage small intestinal morphology in rats [17]. Therefore, in our study, a single low dose of octreotide was selected to study its influence on hepatic steatosis associated with obesity [13, 18]. Food intake was measured daily in all groups to calculate the energy intake during the final 8 days.

The rats were fasted for 12 hours and anesthetized with 2% sodium pentobarbital administered intraperitoneally at the end of the experiments. The liver and abdominal adipose (perirenal, testicular, and omental adipose tissue) were collected and weighed. Portions of the liver were either frozen at -80°Cfor the analysis of gene and protein expression or fixed in 4% paraformaldehyde for further histopathological analysis. The blood samples were centrifuged (1,000 rpm, 4°C, 15 min) to separate the plasma and then stored at -80°C for use.

The Institutional Animal Care and Ethic Committee of Sichuan University (Approved No. SYXK 2008–119) approved all animal studies.

### Fasting glucose, lipids, insulin, SST and HOMA index

The fasting plasma glucose (FPG) concentration was determined with a blood glucose assay (Maker Science And Technology Co., Sichuan, China). The fasting serum insulin was determined with a radioimmunoassay kit (Millipore Co., Billerica, MA, USA). To estimate the insulin sensitivity, the homeostatic model assessment (HOMA) value was calculated as previously described [19]. The serum TG and total cholesterol levels (TC), low-density lipoprotein cholesterol (LDL-C) and high-density lipoprotein cholesterol (HDL-C) were measured using enzymatic methods (ZhongSen Co., Beijing, China), respectively. Free fatty acid (FFA) was also measured (Randox, Crumlin, UK). The SST levels were measured with an SST radioimmunoassay kit (RIA Technology Development Center, PLA General Hospital, Beijing, China). In this study, radioimmunoassay (RIA) was performed to measure the SST levels. However, RIA could not distinguish between octreotide and its analog SST due to their similar oligopeptide structure. In the preliminary test for SST measurement, octreotide competitively replaced the binding of ^125^I-SST with its antibody. Therefore, the total binding to SST antibody in the plasma after octreotide injection was defined as SST-like immunoreactivity (SST-LIR) [20].

### Histopathological study of liver steatosis

Liver tissues were fixed, dehydrated and embedded in paraffin. Paraffin sections (3-μm thickness) were then subjected to hematoxylin and eosin (HE) staining. The liver tissue morphology and unstained areas of fat vacuoles were observed under a light microscope. Oil red O (ORO) staining was performed to detect neutral lipids and lipid droplet morphology with slight modifications [21]. Briefly, rat livers were flash-frozen in liquid N_2_ and subjected to frozen sectioning. Then, the frozen tissue sections (9-μm thickness) were immersed in 1% oil red O working solution for 10 min, counterstained with hematoxylin, and then rinsed under running tap water for 30 min. Photomicrographs were captured under a microscope (Olympus, Tokyo, Japan) from different views. The integrated optical density (IOD) of the oil red O-stained area was analyzed with Image-Pro Plus 6.0 software (Media Cybernetics, Silver Spring, MD, USA).

### Liver lipid content

To determine the liver lipid content, hepatic lipids were extracted [22]. Briefly, liver tissues (15 mg) were homogenized in 1 ml of chloroform: methanol solution (v/v, 2:1), shaken for 20 min and centrifuged at 1800 rpm for 10 min. The supernatant was separated, mixed with 0.9% NaCl and centrifuged at 2000 rpm for an additional 20 min. The organic phase was dried and re-dissolved in 3% Triton X-100. After lipid extraction, TG concentration in the liver was measured with commercial kits (ZhongSen Co., Beijing, China), which are presented as milligrams of triglyceride per gram of liver.

### Hepatic VLDL production

In a separate experiment, the rats from three groups (control, obese and octreotide-treated group) were fed as described above. At the end of the octreotide treatment, three groups of rats were fasted for 12 h and injected with Triton WR-1339 (Sigma-Aldrich, USA) via the tail vein at a dose of 500 mg/kg body weight [23,24]. Plasma samples were collected at 0, 1, 2, 3, and 4 h respectively after the injection via tail bleed, and the serum TG was measured using enzymatic methods (ZhongSen Co., Beijing, China). Because Triton WR-1339 effectively blocks the degradation of VLDL [25], the increase in TG represents the VLDL production from the liver.

A linear relationship was observed between VLDL-TG concentrations and time after i.v. injection of Triton WR-1339, and the VLDL-TG secretion rate was calculated as the slope of this linear curve [23].

### RT- PCR

The total RNA from 10 mg of liver tissue was isolated using Trizol reagent (Takara Bio-Engineering Co., Ltd., Kyoto, Japan) following the manufacturer’s instructions. The concentration and purity of the RNA samples were then measured. The RNA was reverse-transcribed into cDNA by using reverse transcription kits (MBI, Fermentas Life Sciences Inc., Vilnius, Lithuania) and then stored at -80°C. The cDNA template was then used to analyze the MTP, ACC1 and apoB mRNA levels via polymerase chain reaction (PCR). Intron-spanning primers were synthesized by the GeneWeiZ biotechnology company of Suzhou China and were listed in Table 1. The PCR products were subjected to electrophoresis in a 1.5% agarose gel, and pictures were obtained with a gel imaging analysis system (BIO-RAD Laboratories Inc., CA, USA). The gray intensity was analyzed using Quantity One software 4.6.2 (Bio-Rad Laboratories, Hercules, CA, USA). GAPDH was used as the internal control.

**Table 1:** List of primers for RT-PCR or qRT-PCR.

Gene	GenBank Accession No.	Forward sequence(5’-3’)	Reverse sequence(5’-3’)	Expected Product Size(bp)
GAPDH	NM_017008.4	TCGGTGTGAACGGATTTG	CTCAGCCTTGACTGTGCC	317
ACC1	NM_022193.1	TGAGGAGGACCGCATTTATC	AAGCTTCCTTCGTGACCAGA	221
MTP	NM_001107727.1	CTATCCACAGGGAGGGGC	CCAGGGGAATCAAAACCA	181
ApoB	NM_019287.2	TGGACAGTGAAATATTATGAA	TGGACAGGTCAATCAATCTT	388
SREBP-1c	NM_001276707.1	GCTCACAAAAGCAAATCACT	GCGTTTCTACCACTTCAGG	140
FAS	NM_017332.1	CTATTGTGGACGGAGGTATC	TGCTGTAGCCCAGAAGAG	129
ADRP	NM_001007144.1	CTCTCGGCAGGATCAAAGAC	CGTAGCCGACGATTCTCTTC	171
β-actin	NM_031144.3	CGAGTACAACCTTCTTGCAGC	CCTTCTGACCCATACCCACC	209

ACC1, Acetyl-CoA carboxylase; MTP, Microsomal triglyceride transfer protein; ApoB, apolipoprotein B; SREBP-1c, sterol regulatory element binding protein-1c; FAS, fatty acid synthase; ADRP, Adipose Differentiation-Related Protein.

### Quantitative real-time PCR (qRT-PCR)

The 2×SYBR Green qPCR Master Mix qRT-PCR RNA Detection Kit (Biotool, Houston) was used in conjunction with SYBR Green for the quantification of the ADRP, FAS, and SREBP-1c genes. Total RNAs from the liver tissues were extracted using Trizol reagent (Takara Bio-Engineering Co., Ltd., Kyoto, Japan). The cDNA was obtained from reverse transcription (RT) reactions. Briefly, ~50 ng of total RNA was subjected to reverse transcription with random primers according to the manufacturer’s instructions. Then, the cDNA products were used for SYBR green-quantitative real-time PCR reaction together with the relative primers (Table 1). The amplification reactions were performed at 95°C for 5 min followed by 35 cycles of 95°C for 15 s and 60°C for 30 s (Biotool, Houston) on the iQ^TM5^ Real Time PCR Detection System. The expression of ADRP, FAS, and SREBP-1c was analyzed from the real-time PCR assay, which was normalized using the 2^-ΔΔCtCt^ method relative to β-actin.

### Western Blot analysis

The concentration of the proteins extracted from the livers was quantitated with a BCA protein assay kit (Pierce Biotechnology Inc., Rockford, IL, United States) prior to mixing with loading buffer and heating at 100°C for 5 min. The extracted protein (40 μg) was separated by SDS-PAGE and then transferred to polyvinylidene difluoride membranes (Millipore, Bedford, MA, USA). Nonspecific binding sites were blocked with 5% non-fat dry powdered milk in incubation buffer, and the membranes were then incubated with antibodies against rat MTP (1:5,000, BD Biosciences, San Jose, CA, USA), SREBP-1c (1:1, 000, Santa Cruz, CA), or ADRP (1:3,000, Epitomics, USA) at 4°C overnight. The membranes were washed, followed by incubation with the appropriate HRP-conjugated secondary antibodies for 2 h at room temperature (Zhongshan Golden Bridge Bio-technology, Beijing, China). Specific bands were visualized by ECL detection. The band densities were quantified using Quantity One software 4.6.2. The housekeeping protein β-actin or GAPDH was analyzed for normalization.

### Immunohistochemical studies of liver sections

After deparaffinization, sections of liver tissue were subjected to high-pressure antigen retrieval for 10 min in citrate buffer at pH 6.0 and treated with 3% H_2_O_2_ for 10 min. After blocking with goat serum, the sections were incubated with anti-FAS (1:100, Bioss, Beijing, China) and anti-ADRP (1:500) overnight at 4°C. The FAS and ADRP proteins were detected by incubating the sections with biotinylated secondary antibodies to form streptavidin-biotin-complexes. Finally, the immunocomplex was visualized by a solution of diaminobenzidine tetrahydrochloride, and the sections were counterstained with hematoxylin. Three fields were randomly selected for each rat liver, and the IOD value was analyzed with Image Pro Plus 6.0 software.

### Enzyme-linked immunosorbent assay (ELISA)

In brief, liver tissue (40 mg) was homogenized at 4°C in phosphate buffered saline (pH 7.4) and centrifuged at 2000 rpm for 20 min at 4°C. The levels of apoB protein were determined using an ELISA kit (R&D Systems, Minneapolis, USA) following the manufacturer’s instructions.

### Statistical analysis

Data are presented as the mean± SD and were analyzed with SPSS 16.0 statistical software (SPSS, Chicago, IL, USA). Comparisons among three groups were performed by one-way ANOVA test, and then Student-Newman-Keuls test was applied to detect significant differences. A test for the homogeneity of variances was also necessary. Differences were considered statistically significant when P values were less than 0.05.

## Results

### Body weight, food intake and parameters associated with obesity

Body weight gain was calculated by the difference between final body weight at the end of the experiments and original body weight in all rats within the 176-day period. As shown in Table 2, the body weight at the 24^th^ week, final body weight, body weight gain and Lee’s index were significantly greater in the obese rats compared to these of the control group (*p*<0.01). The final body weight and body weight gain of the octreotide-treated group were significantly decreased compared to the obese group (*p*<0.05). However, Lee’s index did not differ significantly between these two groups. In addition, octreotide was effective in attenuating the increased abdominal fat and fat/body weight ratio in obese rats.

**Table 2:** The food intake, body weight and parameters associated with obesity in each group.

	Control group (n = 16)	Obese group (n = 16)	Octreotide-treated group (n = 16)
Original Body weight (g)	82.71±6.55	81.59±7.76	84.75±7.38
Body weight at the 24^th^ week (g)	359.00±97.21	597.63±107.82**	599.70±72.46
Final Body weight (g)	377.66±105.86	617.92±136.77**	523.26±105.12^#^
Body weight gain (g)	294.95±104.61	537.00±138.20**	438.51±104.89^#^
Lee’s Index	319.49±20.10	337.57±10.81**	332.52±20.90
Food intake (g/day/rat)	32.89±2.54	28.24±4.29**	27.30±2.04
Energy in chow (kcal/g)	2.90	4.30	4.30
Energy intake (kcal/day/rat)	95.37±7.36	121.45±18.46**	117.40±8.76
Abdominal fat (g)	15.36±8.94	42.70±18.34**	18.53±6.53^##^
Fat/body weight ratio	4.03±1.92	7.02±2.31**	3.61±1.24^##^
Liver weight (g)	11.41±3.03	15.43±4.12**	11.79±1.86^##^

Body weight gain was calculated by the difference between final body weight at the end of the experiments and original body weight. Data are expressed as mean ± SD

** *p*<0.01 vs. control group

^#^ *p*<0.05 vs. obese group

^##^ *p*<0.01 vs. obese group

The consumption of a high-fat diet led to a decrease in food intake (g/day/rat); however, energy intake (kcal/day/rat) in the obese group was 27.3% higher than that in the control group (*p<*0.01). The food/energy intake on the HFD was not significantly different between the obese group and the octreotide-treated group. The octreotide intervention had no effect on food/energy intake in the obese rats.

### Serum glucose, lipids, insulin, SST level and HOMA index

As shown in Table 3, the serum FPG, TG, TC, LDL-C, FFA, insulin and HOMA index were significantly increased in the obese group compared to the control group. Octreotide treatment significantly reduced the levels of these parameters (*p<*0.05). As the good cholesterol, the HDL-C concentration was lower in the obese group compared to that in the control group (*p<*0.05); however, octreotide was not effective in increasing the HDL-C levels. Compared to the control group, the plasma SST levels in the obese group were decreased, but there was no significant difference (*p*>0.05). Nevertheless, the plasma SST levels notably increased in the octreotide-treated group compared to the obese group (*p<*0.05).

**Table 3:** The serum lipid, fasting glucose, insulin, somatostatin levels and HOMA index in each group.

	Control group (n = 16)	Obese group (n = 16)	Octreotide-treated group (n = 16)
Serum lipids levels			
Triglyceride (mmol/L)	0.93±0.20	1.54±0.36**	1.12±0.26^##^
Total cholesterol (mmol/L)	1.74±0.31	2.08±0.40*	1.60±0.35^##^
LDL-C (mmol/L)	0.53±0.07	0.70±0.10**	0.50±0.11^##^
HDL-C (mmol/L)	1.01±0.15	0.84±0.13*	0.91±0.20
FFA/ (μmol/L)	327.33±130.59	754.79±129.21**	533.68±203.48^#^
Fasting glucose (mmol/L)	7.13±0.58	8.64±0.93 **	7.81±1.04^#^
Serum insulin (mmol/L)	17.71±11.43	38.01±23.70**	14.27±10.63^##^
HOMA Index	5.15±3.56	14.19±11.37**	6.38±5.28^##^
Somatostatin (pg/mL)	129.51±47.17	116.12±55.42	185.44±98.55^#^

LDL-C, Low-density lipoprotein cholesterol; HDL-C, High-density lipoprotein cholesterol; FFA, Free fatty acid; Data are expressed as mean ± SD

* *p* <0.05 vs. control group

** *p*<0.01 vs. control group

^#^ *p*<0.05 vs. obese group

^##^ *p*<0.01 vs. obese group

### Octreotide reduces liver steatosis in obese rats

**Fig 1:**
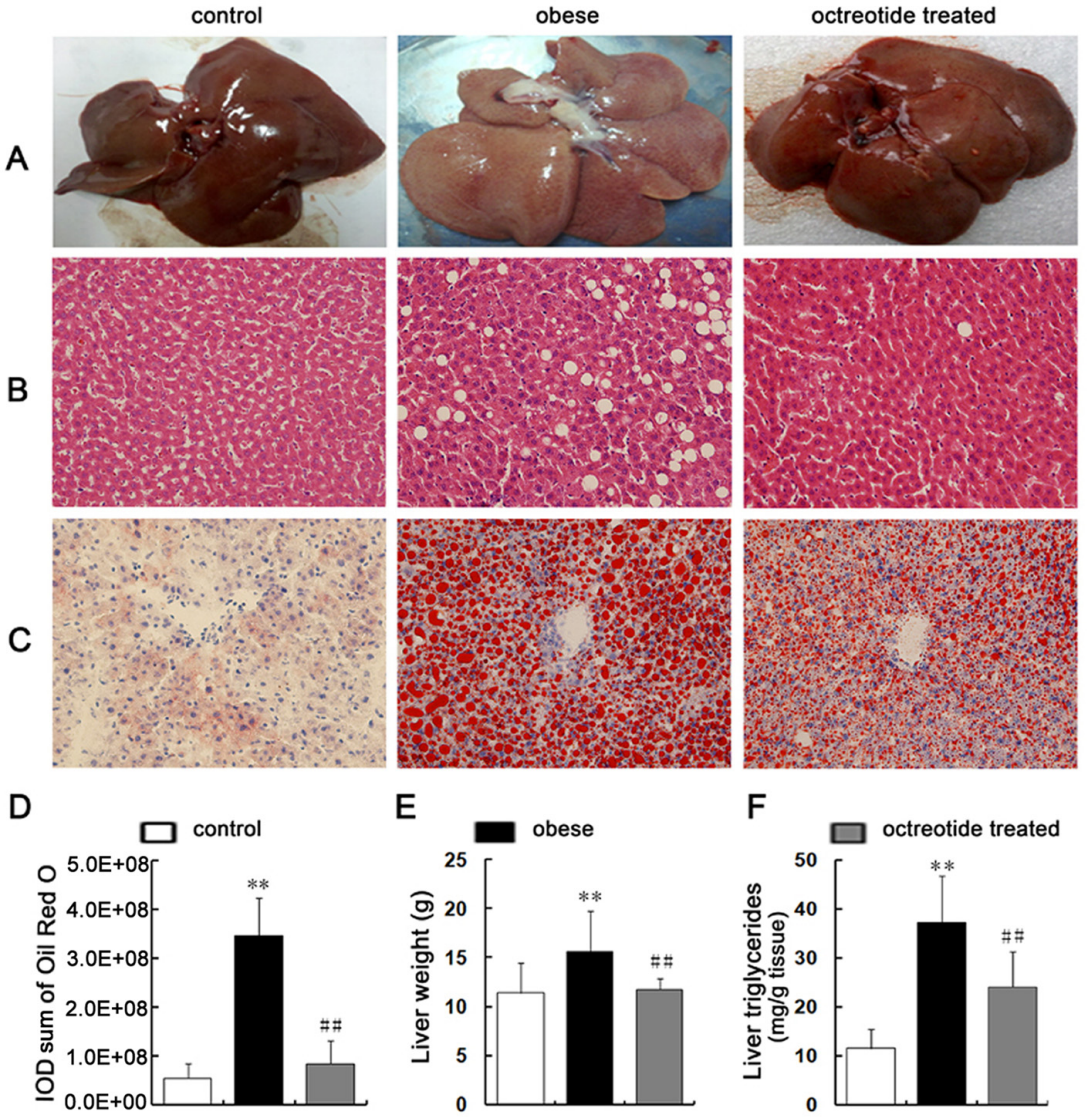
Reduction of hepatic steatosis with octreotide treatment. Compared to the livers in the control group, typical hepatic steatosis was evidenced by a faint yellow color (A), a larger unstained area that consisted of fat vacuoles (B; H&E-stained; ×400 magnification), a larger red area that consisted of lipid droplets (C, D; oil red O staining; ×400 magnification), heavier livers (E), and higher TG contents (F) in the livers of the obese group. Octreotide treatment significantly reversed these changes in the liver. **p<0.01 vs. control group; ##*p<*0.01 vs. obese group.

It can be seen from Fig 1A that the livers of obese rats were slightly yellow in appearance. The liver weights of the obese rats were heavier than that of the control group (Table 2, Fig 1E). By contrast, the livers of the octreotide-treated and control groups were similar in weight and macroscopic appearance. Lipid accumulation was elevated in the obese rats, as indicated by larger H&E unstained areas of hepatocytes (Fig 1B), as well as more red hepatocytes and higher IOD values (*p<*0.01; Fig 1D) in the oil red O-stained sections (Fig 1C) compared to the control group. However, octreotide treatment significantly decreased the H&E unstained area and the oil red O-stained red tissue. Also, the IOD value of the octreotide-treated group was significantly lower than that of the obese group (*p<*0.01). Consistent with these results, the TG content in the liver tissue was 3.2 times higher in the obese rats than in the normal control group, whereas the levels of liver TG decreased by 35.5% after octreotide treatment (Fig 1F).

### Expression of hepatic genes and proteins for fatty acid synthesis

**Fig 2:**
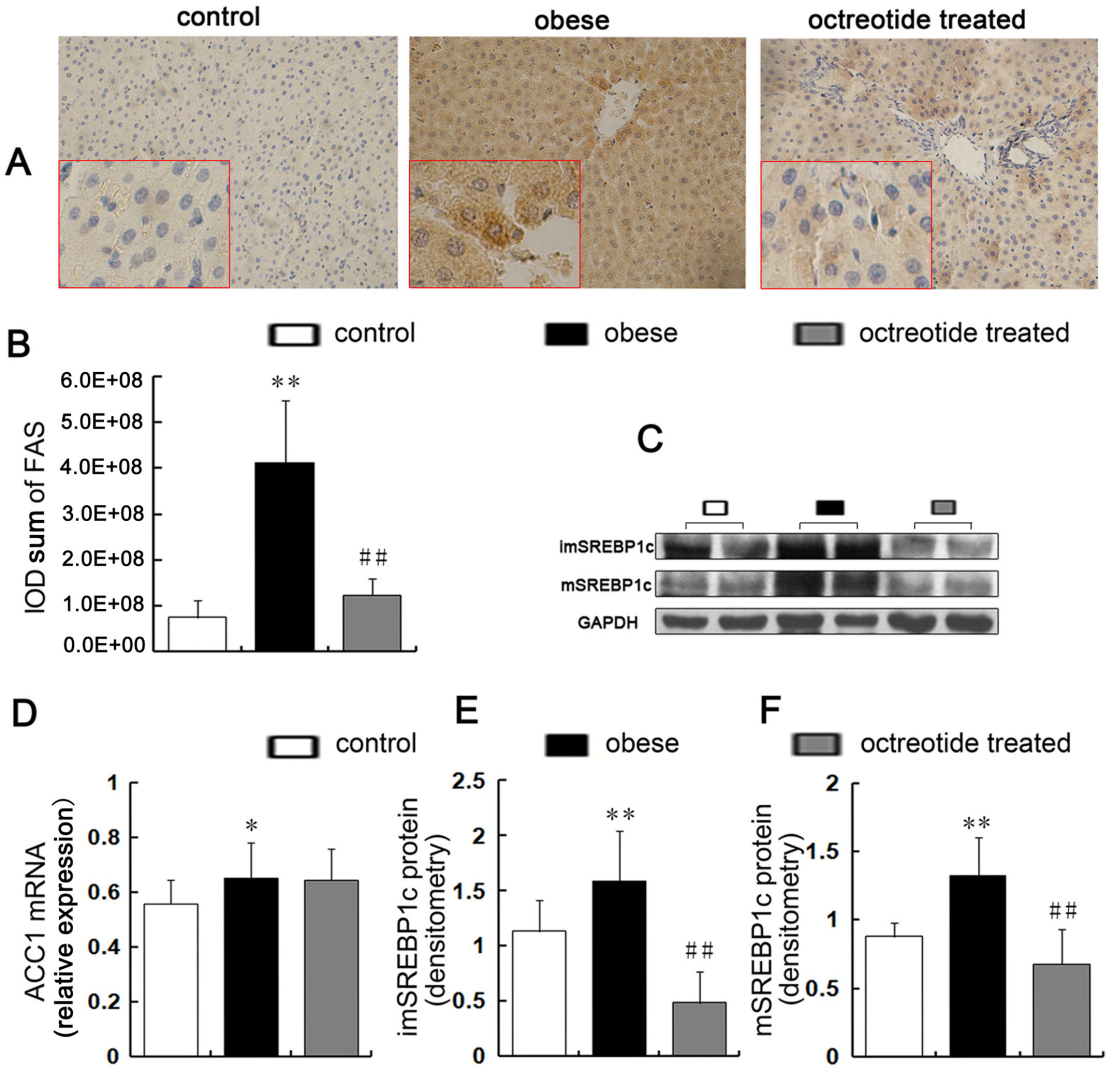
Expression of key factors related to hepatic lipogenesis synthesis. The positive expression of FAS is indicated by brown granules (A, immunohistochemistry stain, ×400 magnification). The protein levels of FAS were detected by immunohistochemistry (A, B). Immature SREBP1c (C, E) and mature SREBP1c (C, F) were measured by western blot and were significantly higher in the obese group than the control group, whereas octreotide intervention inhibited these increases to varying degrees. A semi-quantitative RT-PCR analysis showed that the level of ACC1 mRNA was higher in the obese group than the control group, but the liver ACC1 mRNA levels did not differ between the obese and octreotide-treated groups (D). *p<0.05 vs. control group; **p<0.01 vs. control group; # *p<*0.05 vs. obese group; ## *p<*0.01 vs. obese group.

**Fig 3:**
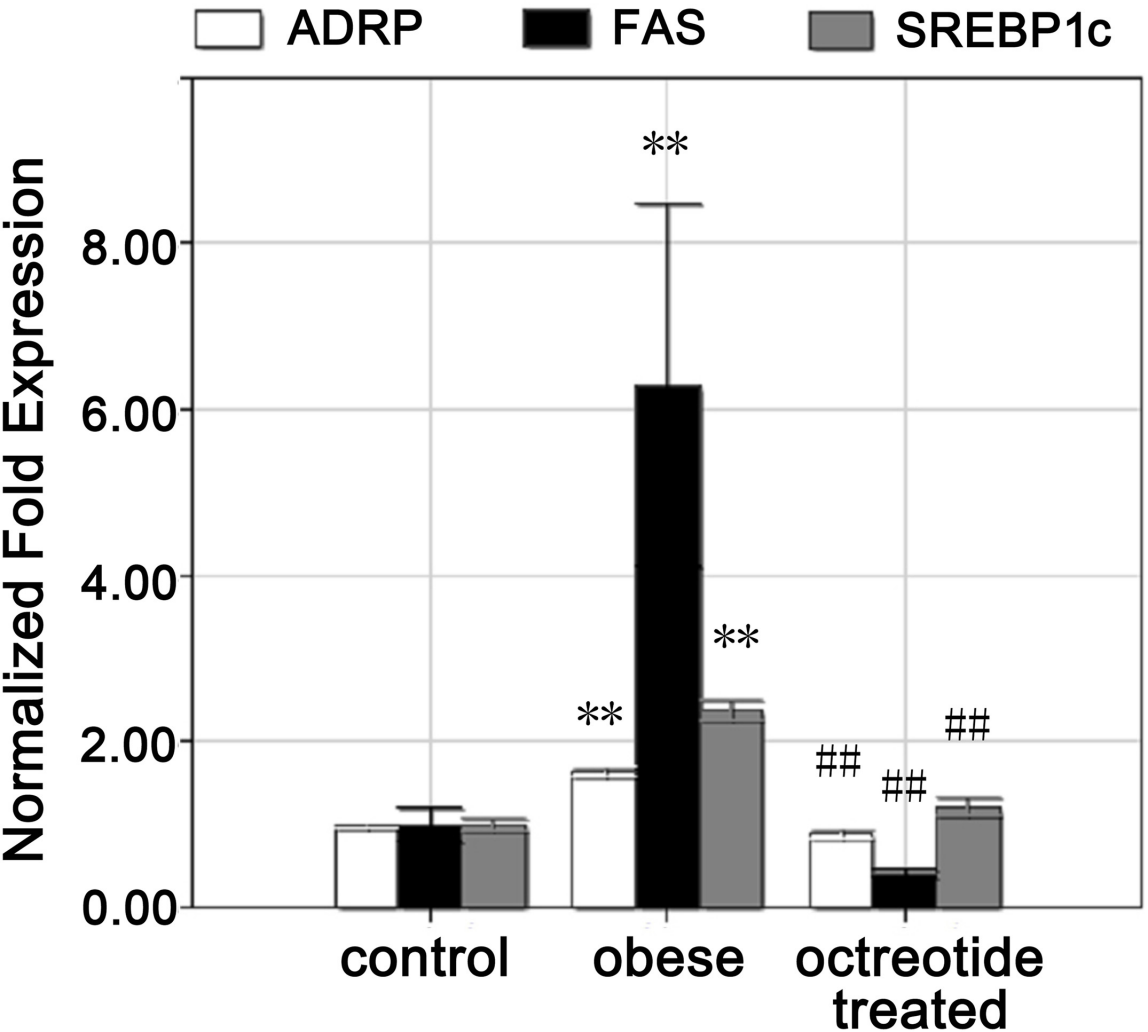
Expression of SREBP1c, FAS and ADRP mRNA in the liver. The quantitative real-time PCR showed that the levels of SREBP1c, FAS and ADRP mRNA in the obese group were higher than the control group, whereas octreotide intervention inhibited the expression of these genes. **p<0.01 vs. control group; ##*p<*0.01 vs. obese group.

Both immature and mature SREBP-1c proteins (Fig 2C, 2E and 2F) were elevated in the obese group compared to the control group (*p*<0.01), but octreotide treatment induced decreases of immature and mature SREBP-1c proteins by approximately 69.3 and 48.8% (*p*<0.01), respectively. The results of the immunohistochemical staining showed that the up-regulation of FAS in the livers of the obese group significantly decreased in response to octreotide treatment (Fig 2A and 2B). Fig 3 shows that compared to the control group, the obese group had a 2.4-fold higher SREBP-1c level and 6.3-fold higher FAS level, respectively. However, treatment with octreotide led to a significant decrease in SREBP-1c and FAS expression (48.6 and 93.3%, respectively) in the octreotide-treated rats, the results of which were similar to the control group. The liver mRNA levels of ACC1 increased by 19% in the obese group compared to the control group (*p*<0.05). Unexpectedly, the liver ACC1 mRNA levels in the obese and octreotide-treated groups showed no difference (*p*>0.05; Fig 2D).

### Effect of octreotide on hepatic VLDL production

**Fig 4:**
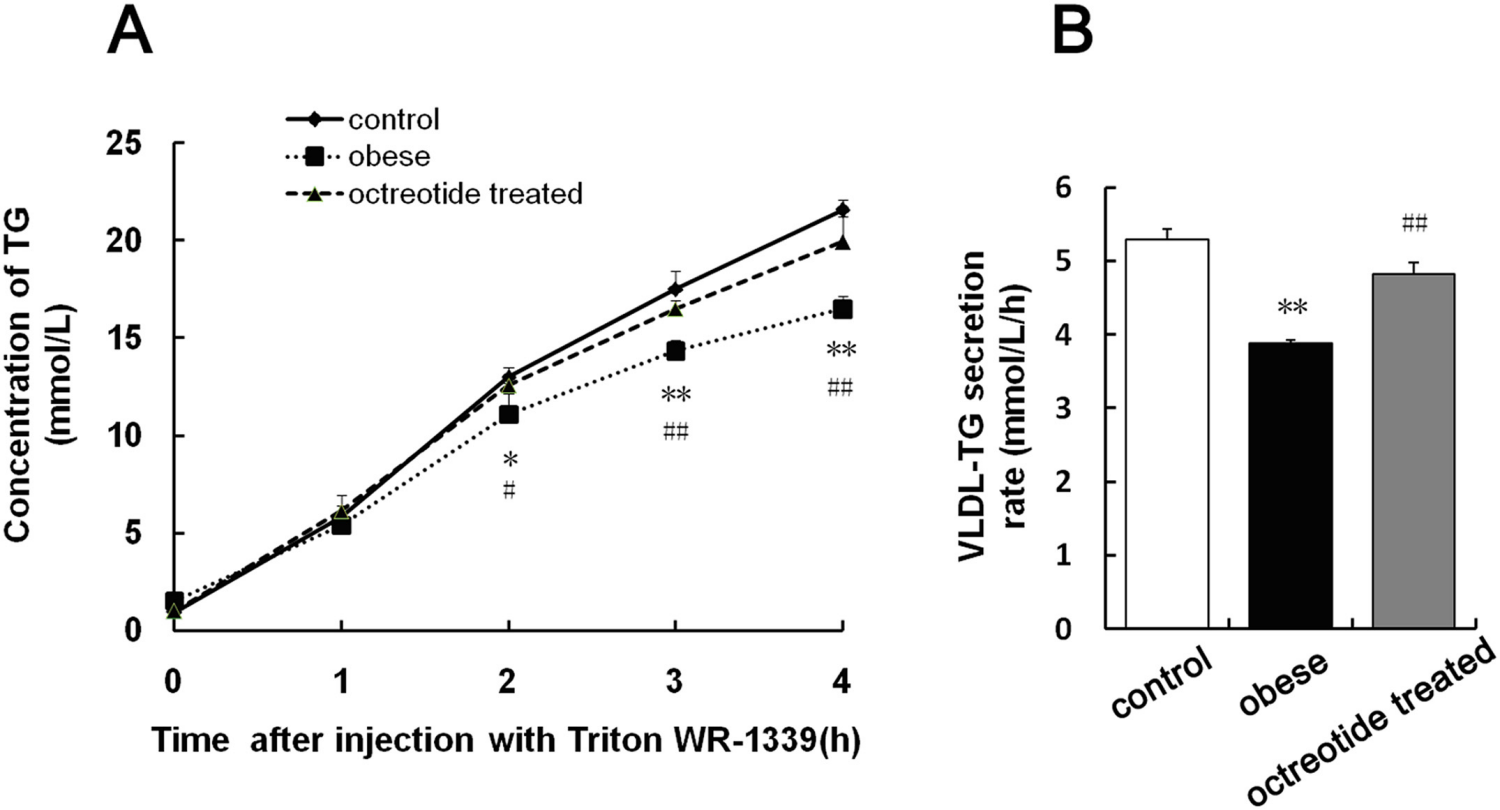
Hepatic secretion of VLDL-triglycerides in fasting rats after i.v. administration of Triton WR-1339. Three groups of rats (control, obese and octreotide-treated group) were fasted for 12 hours prior to tail intravenous injection of Triton WR1339 at 500 mg/kg body weight. Blood samples were taken at 0, 1, 2, 3, and 4 hours post-injection for the estimation of plasma TG concentrations (A). The VLDL secretion rates (mmol/L/h) were calculated using the slopes of the curves (B). Data are presented as the mean ± SD (*n* = 3). *p<0.05 vs. control group; **p<0.01 vs. control group; #*p<*0.05 vs. obese group; ##*p<*0.01 vs. obese group.

Fig 4A shows that the TG concentration was increasing as function of time in all groups. Also, compared with obese group, octreotide could significantly induce TG secretion after 2-h Triton WR-1339 injection (*p*<0.05 at 2 h; *p*<0.01 at 3 and 4 h). To our best knowledge, the VLDL-TG secretion rate was calculated as the slope of the curve linearization between VLDL-TG concentrations and time [23]. The VLDL-triglyceride secretion rate in the obese group was significantly decreased compared with the control group (*p*<0.01; Fig 4B). Treatment with octreotide resulted in a significantly increased hepatic VLDL-triglyceride secretion rate compared to the obese group (*p*<0.01; Fig 4B).

### Expression of hepatic genes and proteins for VLDL assembly and maturation

**Fig 5:**
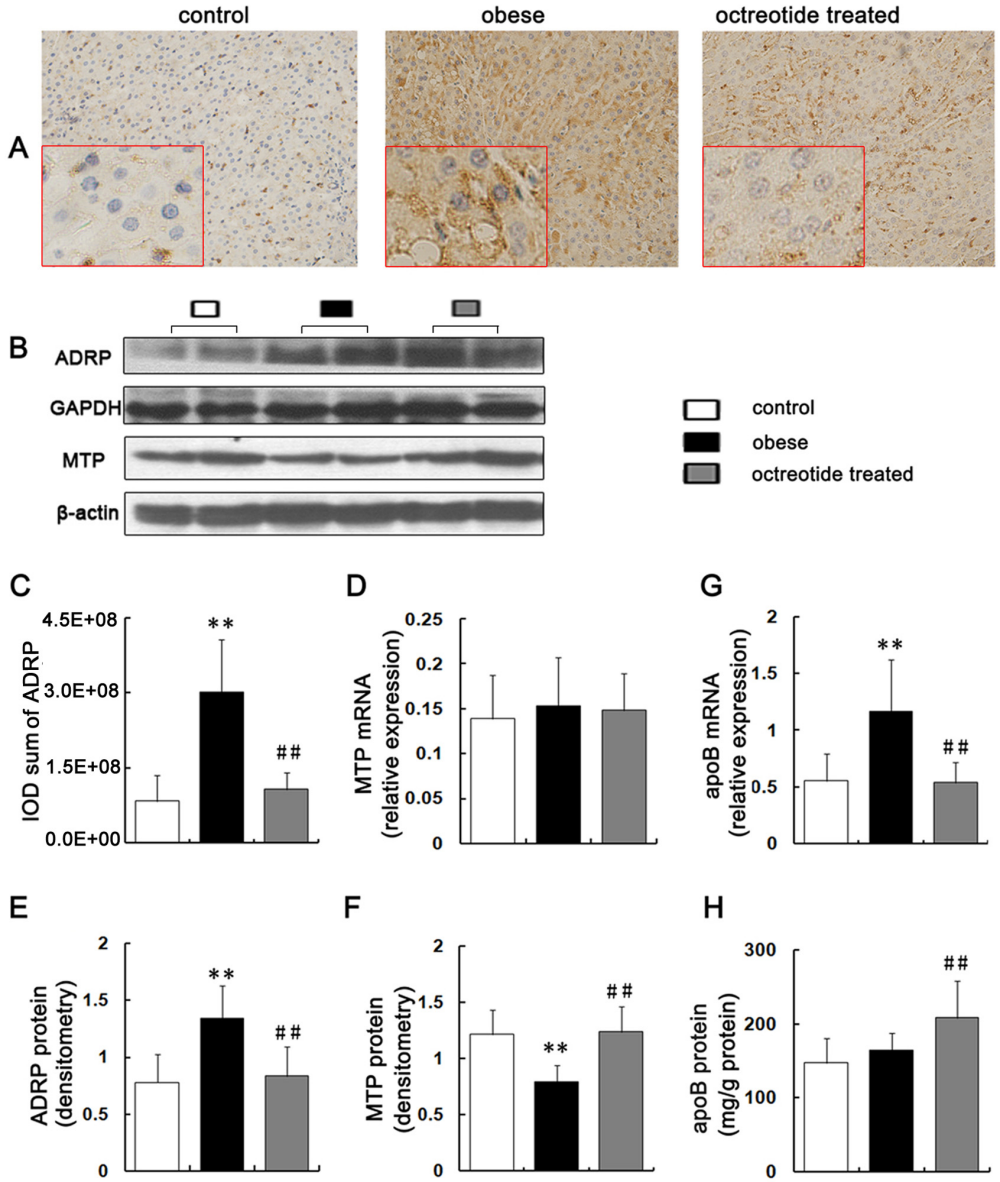
Expression of key factors involved in VLDL assembly, esterification and secretion. The positive expression of ADRP is manifested by brown granules (A, immunohistochemistry staining, ×400 magnification). The Integral Optical Density (IOD) of ADRP were analyzed by Image Pro Plus 6.0 software (C). ADRP expression was significantly higher in obese rats than in the control and octreotide-treated rats, as evidenced by immunohistochemistry and western blot analyses (B, E). The level of MTP mRNA were detected by RT-PCR (D). The protein level of hepatic MTP in the obese group was the lowest of the three groups, as measured by a western blot (B, F). Semi-quantitative RT-PCR showed that the level of apoB mRNA in the obese group was the highest among the three groups (G). The protein levels of apoB were measured by ELISA (H). **p<0.01 vs. control group; ##*p*<0.01 vs. obese group.

The MTP mRNA levels showed no difference among the three groups (*p*>0.05; Fig 5D); however, the hepatic MTP protein levels in the obese rats were decreased compared to the control group. On the contrary, the MTP protein level was significantly increased in response to octreotide treatment (*p*<0.01; Fig 5B and 5F).

RT-PCR showed that the level of apoB mRNA in the obese group increased by 110% and 115.5% compared to the control and the octreotide-treated groups, respectively (*p*<0.01; Fig 5G). In contrast to the mRNA changes, ELISA demonstrated that the apoB protein levels did not differ significantly between the control and obese groups (*p*>0.05). However, the apoB protein level in the octreotide-treated group was significantly increased by 27% compared to the obese group (*p*<0.01; Fig 5H).

The immunohistochemistry indicated that the ADRP protein localized to the surface of the lipid droplets (LDs) (Fig 5A). The immunohistochemical staining (*p*<0.01; Fig 5A and 5C), western blot (*p*<0.01; Fig 5B and 5E) and real-time PCR (*p*<0.01; Fig 3) analyses showed that ADRP protein and mRNA were up-regulated in the obese group compared to the control group. The addition of octreotide significantly down-regulated the levels of hepatic ADRP protein and mRNA (*p*<0.01).

## Discussion

A high-fat diet tends to induce obesity, which increases the prevalence of liver steatosis [26]. In our study, the TG content and lipid droplets in the livers of HFD-induced obese rats were significantly increased, indicating the successful establishment of an animal fatty liver model. Interestingly, octreotide treatment potently suppressed liver lipid accumulation.

Liver steatosis arises from an imbalance between lipid input (fatty acid uptake and de novo lipogenesis) and output (VLDL export and fatty acid oxidation) [4]. SREBP-1c is the key factor for hepatic de novo lipid synthesis and is synthesized as precursors in the endoplasmic reticulum. Then, proteolytic cleavage allows the activation and accumulation of SREBP in the nucleus. Mature SREBP-1c is an activated transcription factor that promotes the expression of key enzymes involved in hepatic lipogenesis, including FAS and ACC1. In this study, mature SREBP-1c, immature SREBP-1c, FAS protein and ACC1 mRNA increased in the livers of obese rats compared to the control group. Consistent with these findings, previous studies showed the high expression of FAS and ACC1 in HFD-induced obese mice [27]. It was recently reported that SREBPs are essential for hepatic steatosis in dietary and genetic rodent models of obesity [28], suggesting that a high level of SREBP-1c in high-fat diet-induced obese rats up-regulates the expression of FAS and ACC1 and subsequently increases lipid synthesis, which may be associated with increased insulin levels in obese rats. In fact, insulin can up-regulate and activate hepatic SREBP-1c both transcriptionally and post-translationally, irrespective of insulin resistance [29]. Studies reported that somatostatin and somatostatin analogs can inhibit insulin release via the receptor-selective direction of somatostatin receptor subtype 5 on the membrane surface of islet B cells [30]. Interestingly, we found that octreotide reversed the HFD-induced up-regulation of insulin, SREBP-1c and FAS in the liver, whereas the transcriptional level of ACC1 remained unchanged. Octreotide is likely to improve insulin resistance in obese rats, which promotes the transport of glucose, accelerates the glycolysis and oxidation of glucose, and increases the level of citric acid. To our best knowledge, citrate is a key inducer for acetyl-CoA carboxylase (ACC) expression and activity [31], and increased citric acid may inhibit the decrease in ACC1 expression induced by SREBP1c. Therefore, the down-regulation of insulin may reduce the transcription of SREBP-1c and inactivate SREBP-1c protein, which subsequently decrease the expression of FAS and suppress fatty acid synthesis.

VLDL is the principal vehicle for the export of endogenous TG in the liver. The assembly and maturation of VLDL are hypothesized to occur as two steps. The first step is the lipidation of apolipoprotein B (apoB) with a few lipids aided by MTP, forming lipid-poor pre-VLDL particles [32, 33]. The second step is the addition of bulk lipids in the microsome to the pre-VLDL particles, forming the lipid-rich mature VLDL [34]. MTP is a rate-limiting factor for VLDL assembly, and the expression of hepatic MTP protein restrains the secretion rate of VLDL [35]. A polymorphism of the MTP gene is associated with the development of NASH [36]. Moreover, VLDL secretion is down-regulated in ob/ob mice, which suffer from seriously hepatic steatosis [37]. In our study, the low expression of MTP protein in the obese group may lead to the decrease in VLDL secretion rate. Chang et al. found that the amount of VLDL-TG decreased during the late stage of NAFLD (at 24 weeks) as opposed to an increase in the VLDL-TG during the late stage of HFD feeding (at 8 weeks) [38]. In contrast to our results, MTP expression in the livers of Wistar rats fed with HFD for 13 weeks is elevated [39]. The hepatic expression levels of MTP were enhanced in NAFLD patients. However, the expression levels of MTP were reduced in the livers with IR or advanced steatosis [40]. There may be several explanations for the differences between these studies, such as differences in the genetic backgrounds of the animals, dietary ingredients, and study lengths. Additionally, Chang et al. found that DNA methylation of the MTP promoter may contribute to its down-regulation in rat NAFLD induced by feeding a HFD for 24 weeks [38].

ApoB is the main structural protein of VLDL. In this study, the expression of apoB mRNA was increased in obese rats compared to the control group, but the expression of the apoB protein did not differ between these two groups. Previous studies of heterozygous MTP knockout mice suggested that half-normal levels of MTP decrease the level of plasma apoB100 to the same extent (25–35%) at any level of apoB synthesis [41]. Therefore, although the expression of apoB protein remains unchanged, low levels of MTP in the obese rat liver can reduce apoB secretion. The overexpression of MTP and apoB protein in the octreotide treatment group can increase VLDL assembly and secretion and subsequently increase hepatic TG output. Interestingly, we observed that octreotide treatment up-regulated the expression of apoB protein and down-regulated the expression of apoB mRNA compared to the obese group. This phenomenon may be attributed to the protection of apoB from degradation due to the normal folding of apoB protein induced by MTP [42], which ensures a higher VLDL secretion rate.

The esterification and maturation processes of VLDL are associated with ADRP. The level of TG in the cytosol is reduced in ADRP-deficient mice, whereas TG concomitantly accumulates in the microsome [43]. The microsomal pool is coupled to VLDL secretion, whereas the cytosolic pool is not [44, 45]. ADRP is suggested to negatively regulate the esterification of VLDL and TG output, and the overexpression of ADRP leads to lipid accumulation in the liver. Previous research showed that ADRP expression is higher in NAFLD than normal livers [46]. The absence of ADRP reduces the amount of TG in the liver and protects the liver against diet-induced fatty liver development [43]. In our study, octreotide treatment reversed the up-regulation of ADRP in high-fat diet-induced obese rats. The down-regulation of ADRP in the octreotide-treated group may positively regulate hepatic microsomal TG accumulation and promote esterification and the secretion of TG-rich VLDL to ultimately reduce the abnormal accumulation of hepatic TG.

In fact, octreotide has been used to cure clinical diseases for several decades, such as pancreatitis and gastrointestinal bleeding, with relative safety including no toxicity to the liver and kidney and no genetic-related side effects. As a bioactive molecule, however, many other functions of octreotide were overlooked. In this study, we found that octreotide improved nonalcoholic fatty liver disease, suggesting that octreotide is a novel promising drug for treating fatty liver disease.

To our knowledge, unsatisfactory safety has limited the practical use of conventional drugs for nonalcoholic fatty liver disease. Unlike conventional drugs, octreotide is an analog of endogenous somatostatin (SST). In addition, octreotide can improve the low level of somatostatin in obesity, improve insulin resistance and pancreatic fat, and inhibit the intestinal absorption of fat. These properties indicate the good biocompatibility of octreotide. Here, we have shown that octreotide decreases rat liver fat levels by regulating SREBP-1c, ACC, FAS, MTP, apoB, and ADRP expression. However, the detailed molecular mechanism remains uncertain. It was reported that the adenosine monophosphate-activated protein kinase (AMPK) pathway plays an important role in modulating hepatic lipogenesis [47, 48, 49]. First, after the administration of octreotide, the activation of AMPK inactivates the ACC protein, which is the rate-limiting enzyme in fatty acid synthesis. Second, activated AMPK impairs mTORC1 signaling that inhibits SREBP-1c, a key transcription factor responsible for fatty acid synthesis. Therefore, we propose that the AMPK pathway is likely to be the molecular mechanism for the octreotide effects, which is currently under study by our group. Taken together, octreotide can ameliorate hepatic steatosis in obese rats fed a high-fat diet and is a promising drug for treating nonalcoholic fatty liver disease.

## Supporting Information

S1 File Original results of the RT-PCR electrophoretic gel analysis for hepatic ACC1 mRNA levels. (DOC)

S2 File Original results of the RT-PCR electrophoretic gel analysis for hepatic ApoB mRNA levels. (DOC)

S3 File Original results of the RT-PCR electrophoretic gel analysis for hepatic MTP mRNA levels. (DOC)

S4 File Original results of the western blot analysis for ADRP protein. (DOC)

S5 File Original results of the western blot analysis for MTP protein. (DOC)

S6 File Original results of the western blot analysis for SREBP-1c protein. (DOC)
