# Supplementary figures and images for: Effect of Octreotide on Hepatic Steatosis in Diet-Induced Obesity in Rats
Source: PLoS One. 2016 Mar 22;11(3):e0152085. doi: 10.1371/journal.pone.0152085 (PMC4803296; doi:10.1371/journal.pone.0152085)

S1 Fig. RT-PCR electrophoretic gel analysis for hepatic ACC1 mRNA level. ACC1 mRNA (221bp), GAPDH mRNA (317bp).


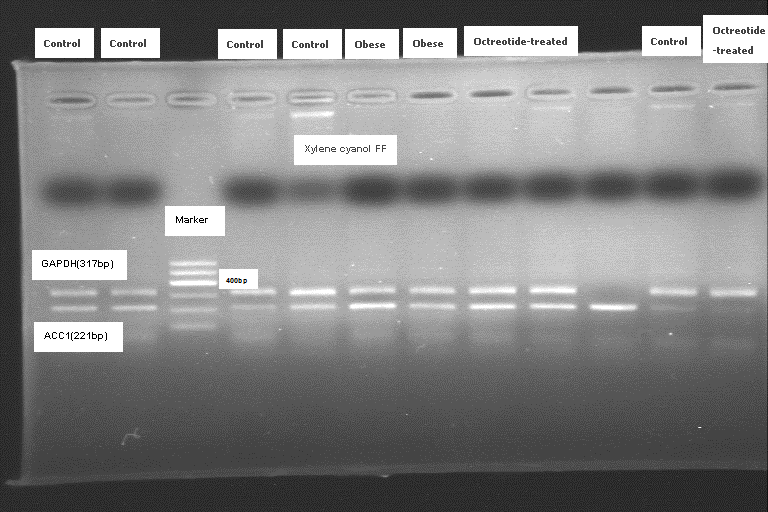


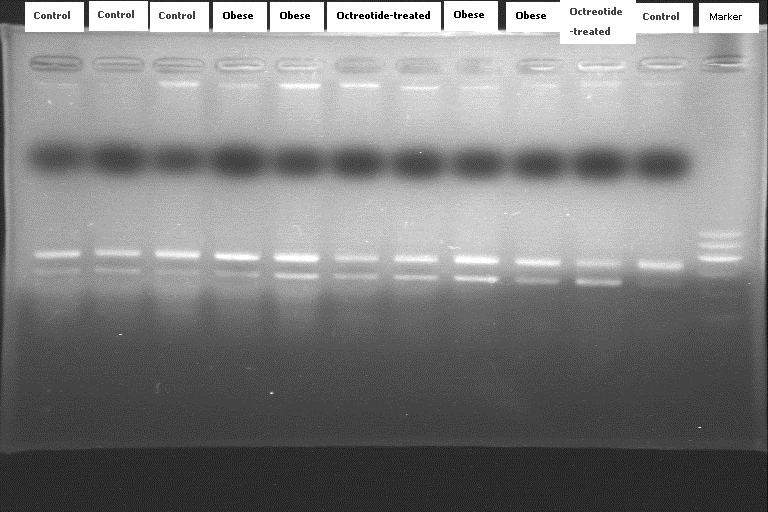


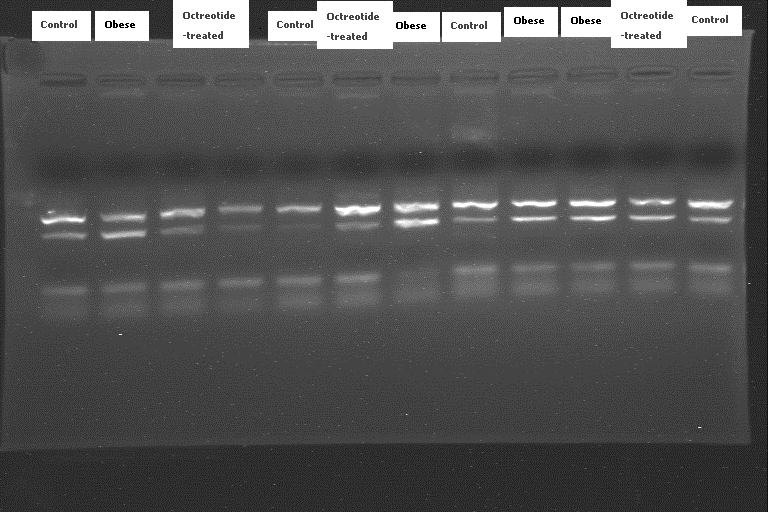


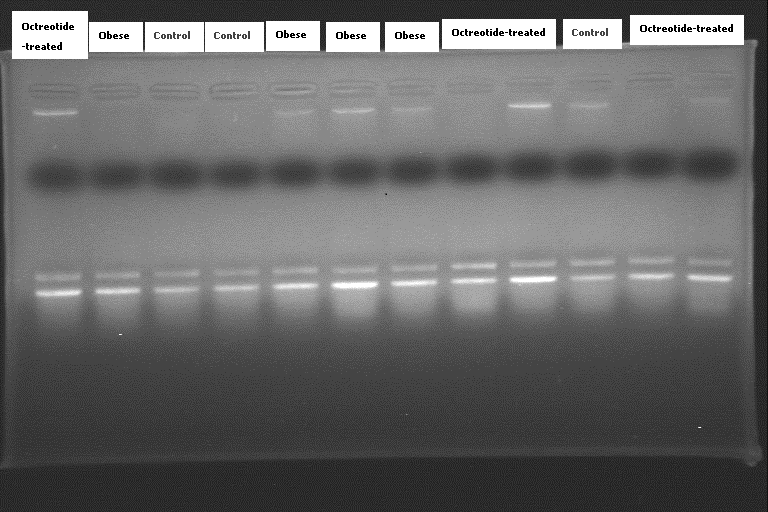


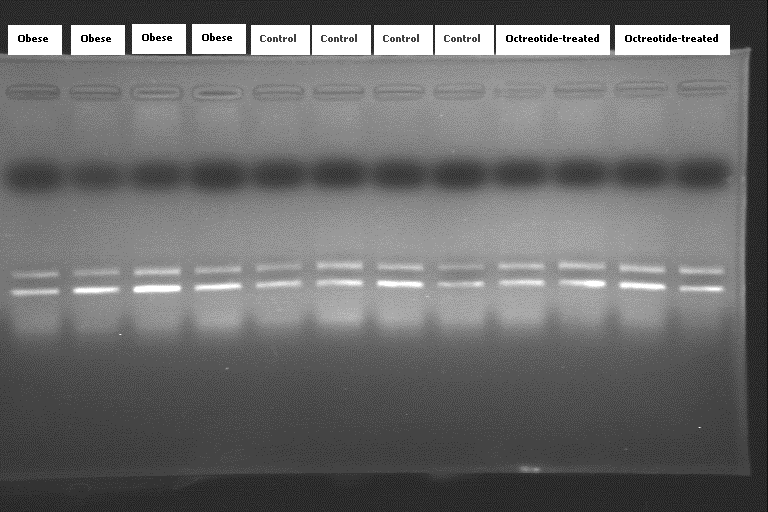

Supplement: S1 File — (DOC) [file pone.0152085.s001.doc]

S2 Fig. RT-PCR electrophoretic gel analysis for hepatic ApoB mRNA level. ApoB mRNA (388bp), GAPDH mRNA (317bp).


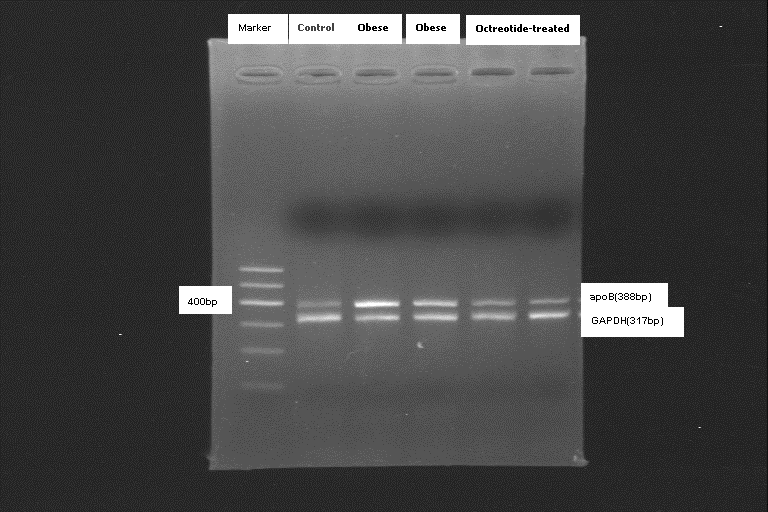


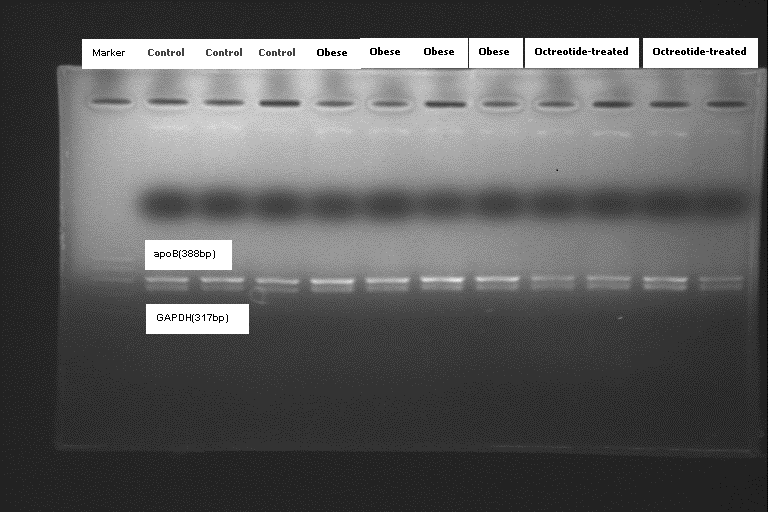


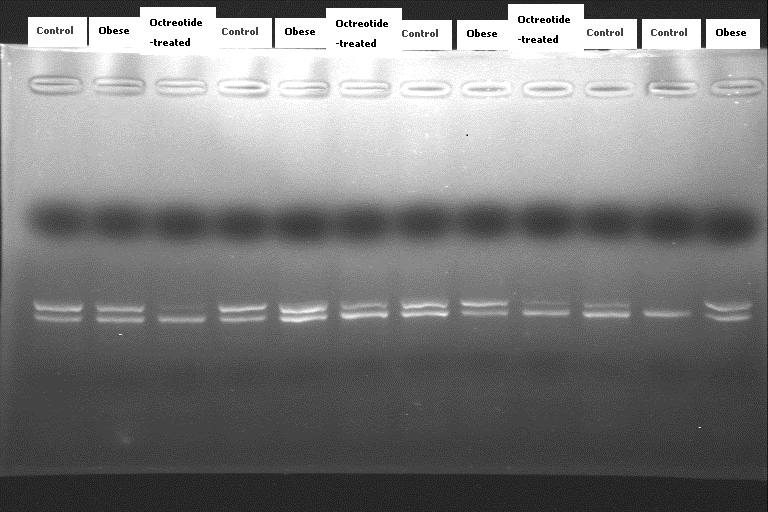


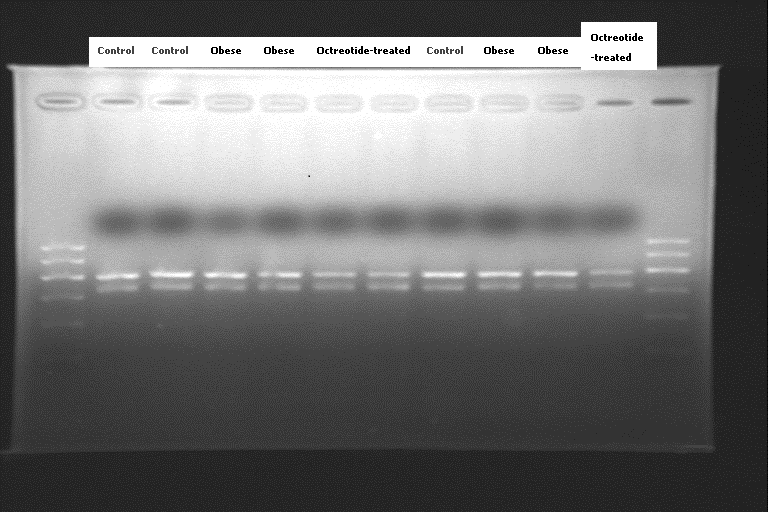


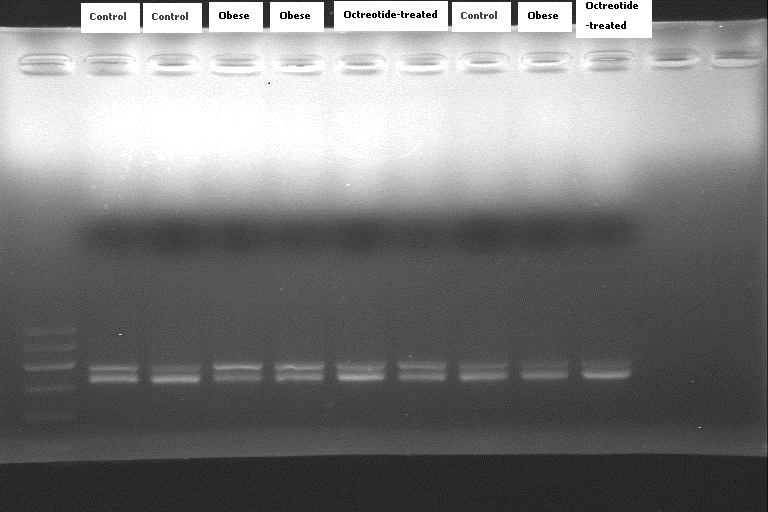


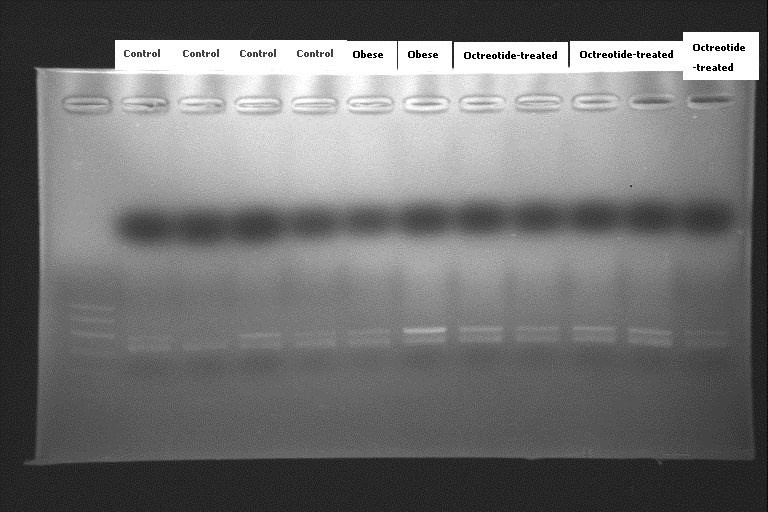

Supplement: S2 File — (DOC) [file pone.0152085.s002.doc]

S3 Fig. RT-PCR electrophoretic gel analysis for hepatic MTP mRNA level. MTP mRNA (181bp), GAPDH mRNA (317bp).


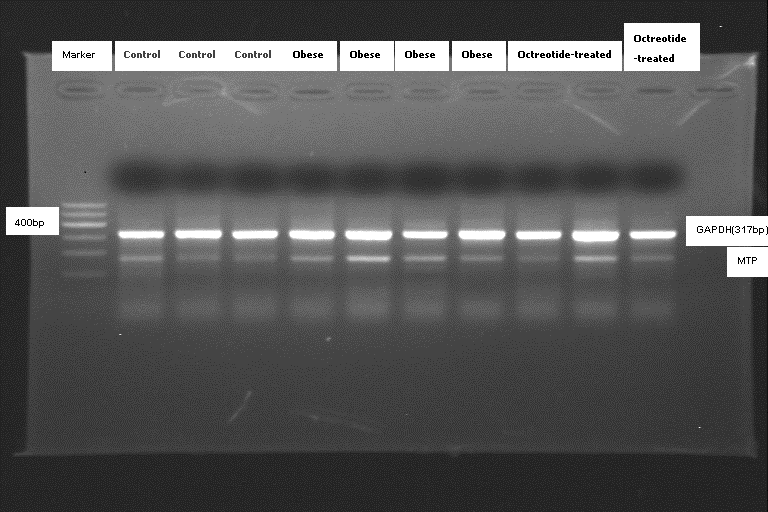


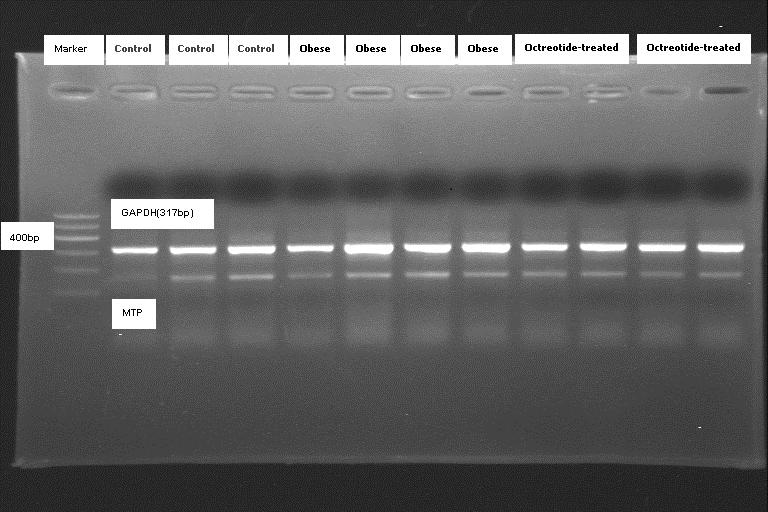


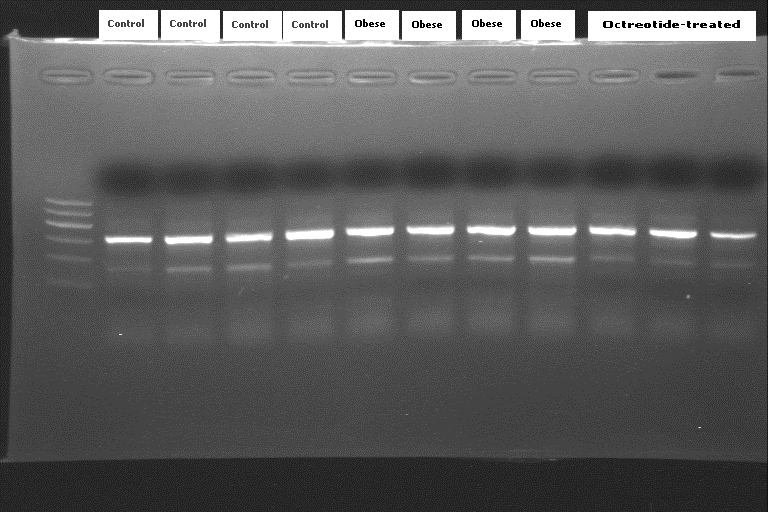


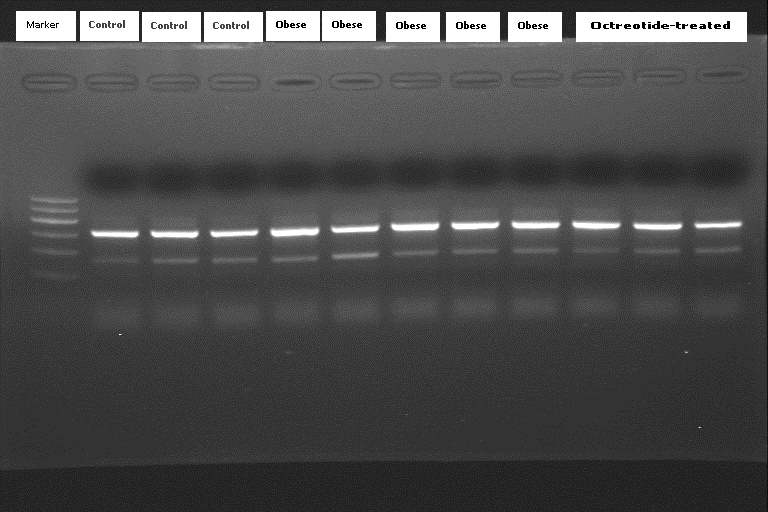


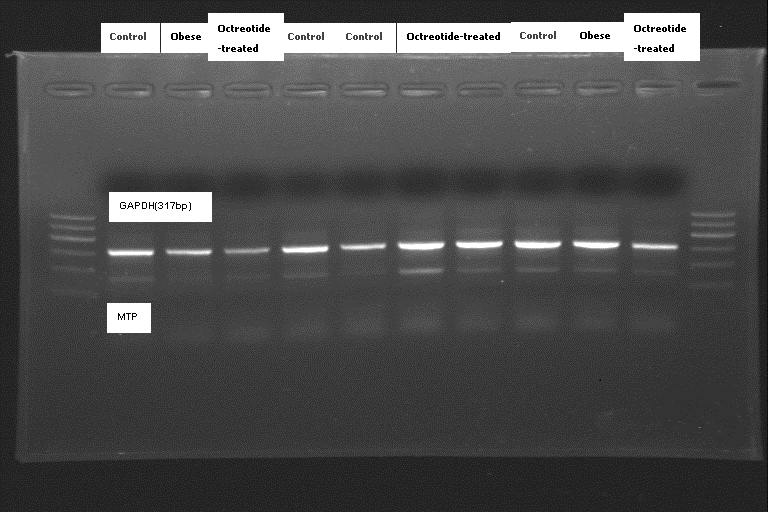

Supplement: S3 File — (DOC) [file pone.0152085.s003.doc]

S4 Fig. Original results of western blot analysis for ADRP protein. ADRP (48KD), GAPDH (37KD).


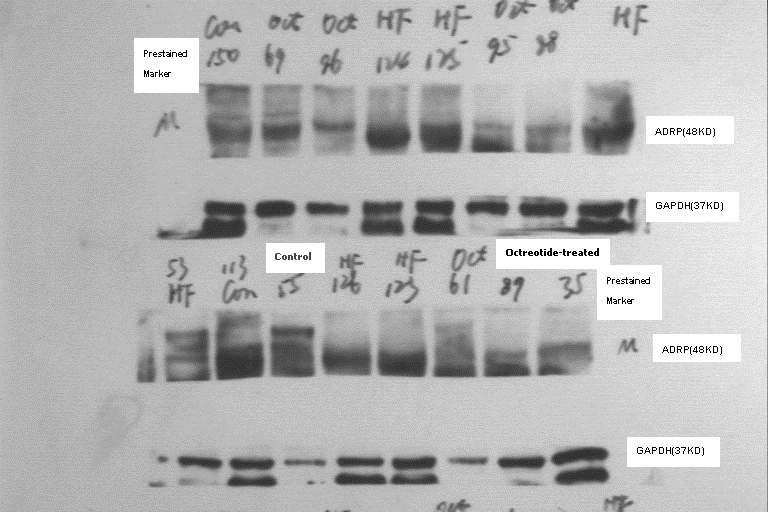


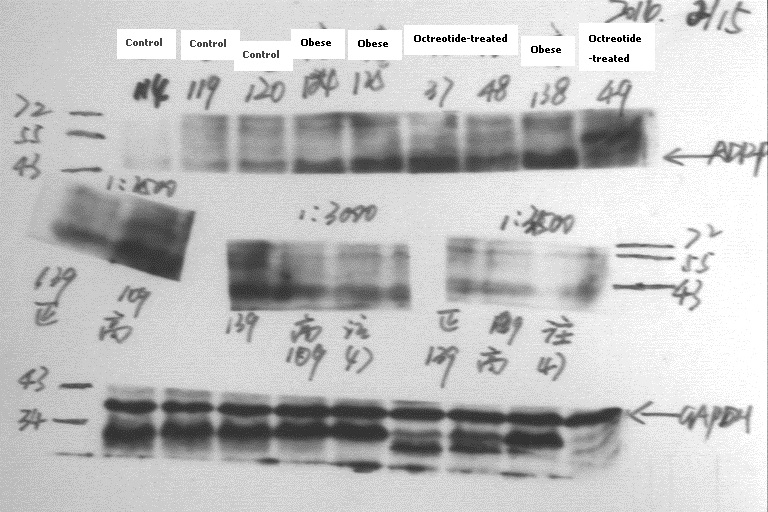


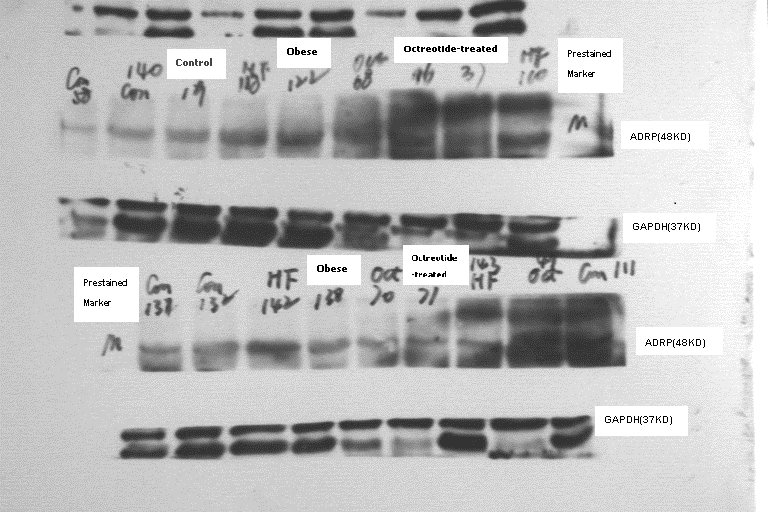


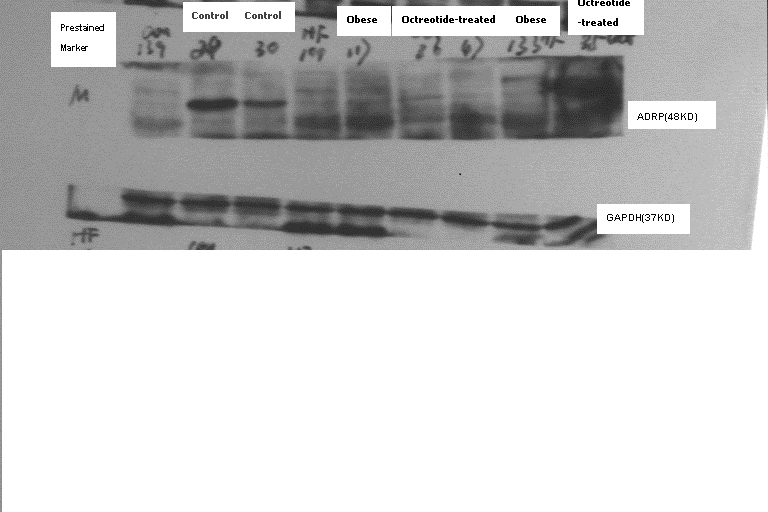

Supplement: S4 File — (DOC) [file pone.0152085.s004.doc]

S5 Fig. Original results of western blot analysis for MTP protein. MTP (97KD), β-actin (43KD).


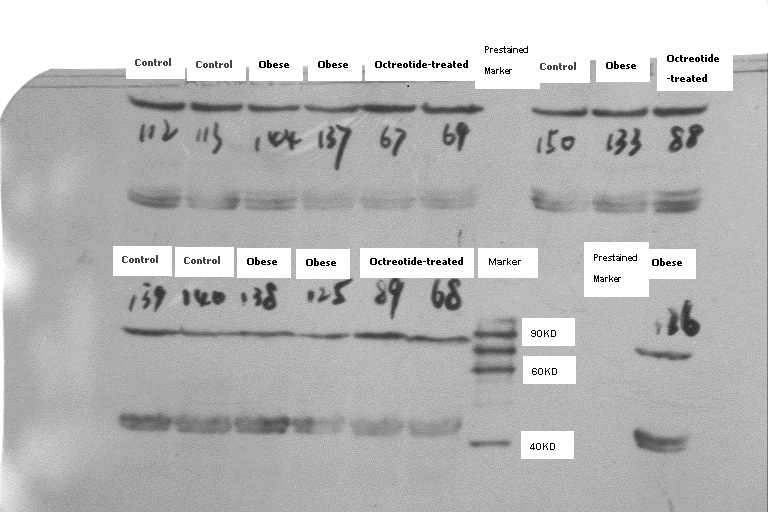


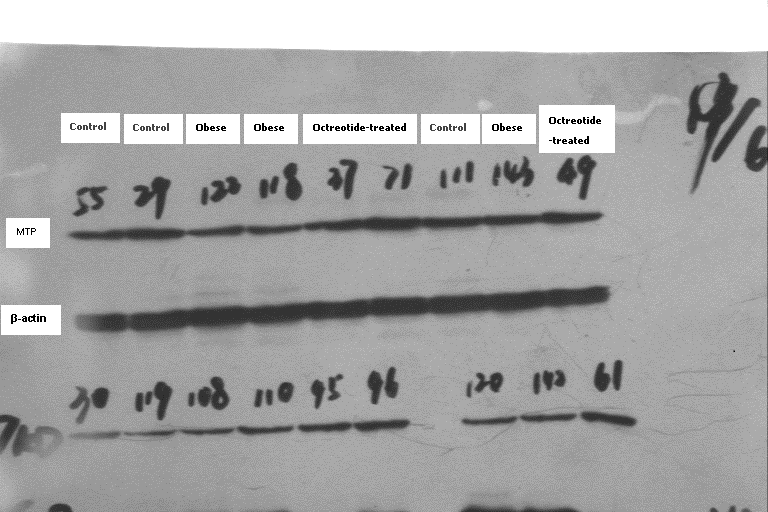


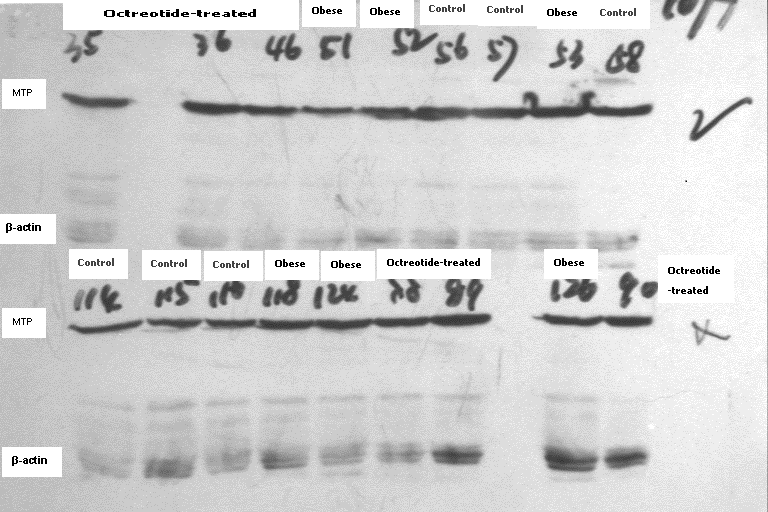


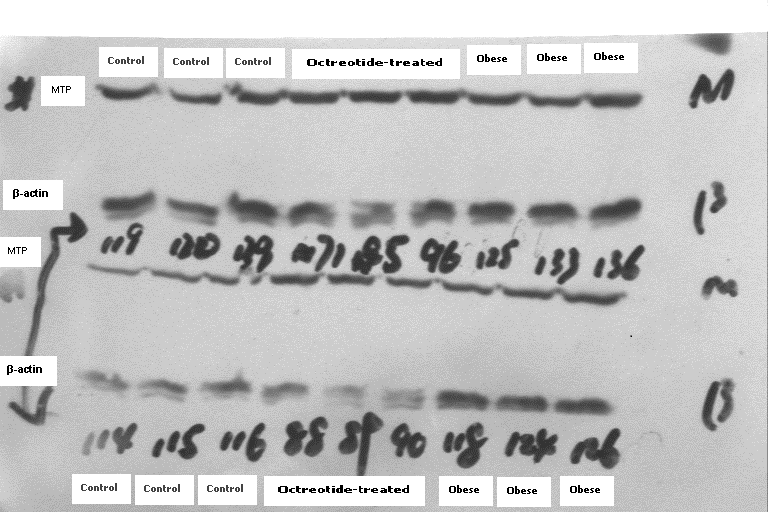

Supplement: S5 File — (DOC) [file pone.0152085.s005.doc]
