# Supplementary material for: Effect of Octreotide on Hepatic Steatosis in Diet-Induced Obesity in Rats
Source: PLoS One. 2016 Mar 22;11(3):e0152085. doi: 10.1371/journal.pone.0152085 (PMC4803296; doi:10.1371/journal.pone.0152085)

S6 Fig. Original results of western blot analysis for SREBP-1c protein. imSREBP-1c (126KD), mSREBP-1c (68KD), GAPDH (37KD).


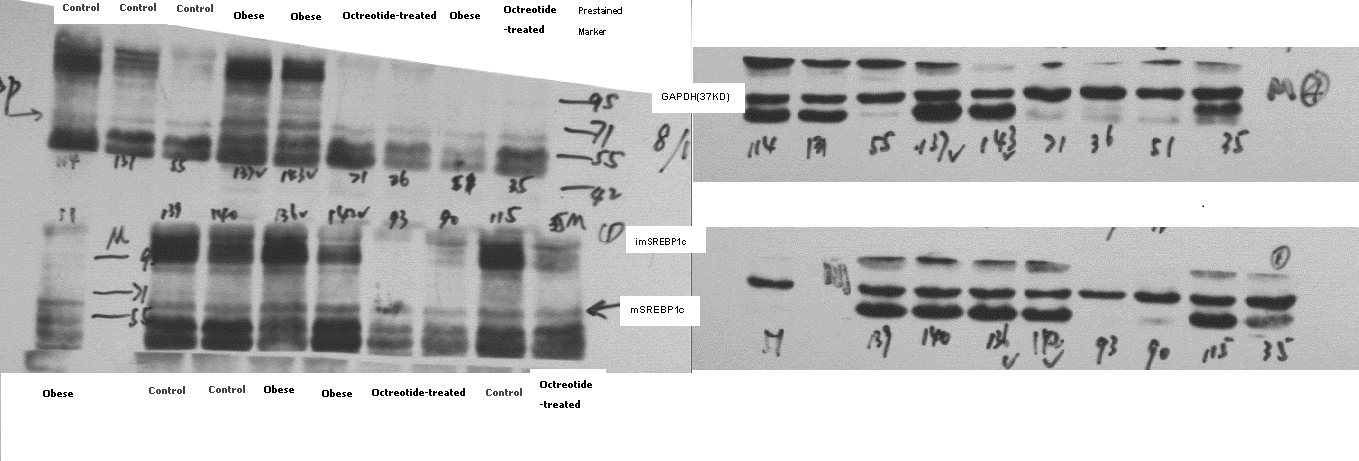


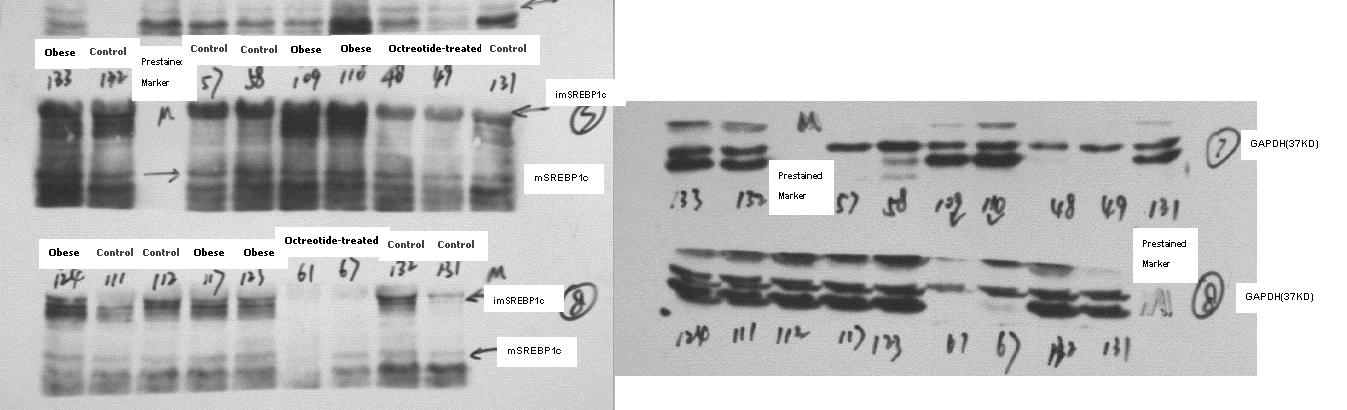


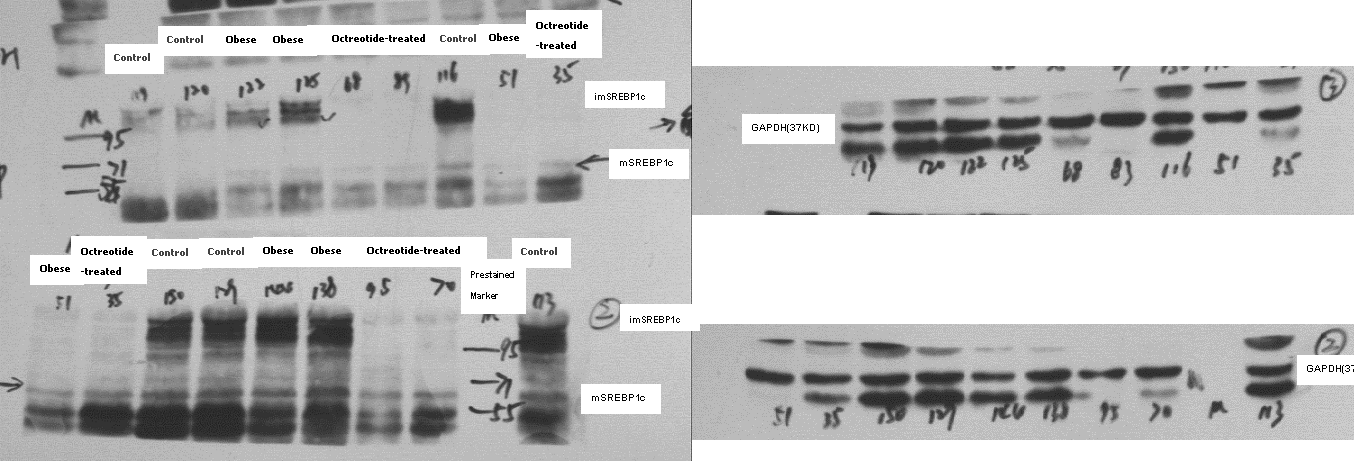


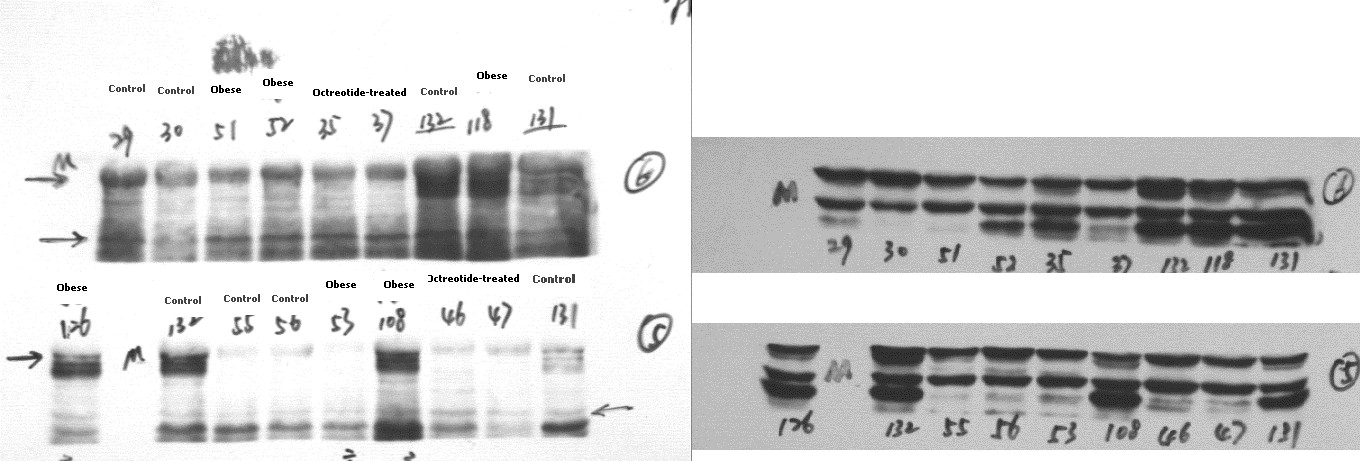


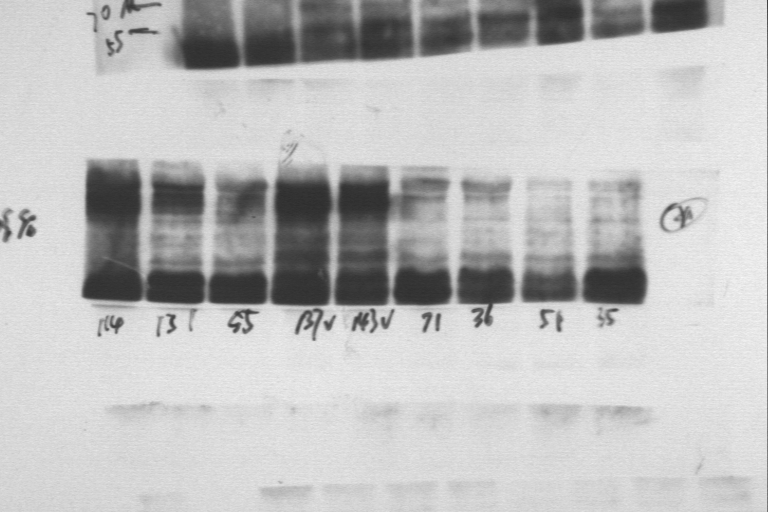

Supplement: S6 File — (DOC) [file pone.0152085.s006.doc]
